# Factors associated with anemia among women of the reproductive age group in Thatta district: study protocol

**DOI:** 10.1186/s12978-019-0688-7

**Published:** 2019-03-18

**Authors:** Sumera Aziz Ali, Zahid Abbasi, Anam Feroz, K Michael Hambidge, Nancy F Krebs, Jamie E Westcott, Sarah Saleem

**Affiliations:** 1 Department of Community Health Sciences, The Aga Khan University, Stadium Road, PO Box 3500, Karachi, 74800 Pakistan; 2 University of Colorado School of Medicine, 12700 E 19th Ave.; Research Complex 2, Rm 5025, Box C225, Aurora, CO 80045 USA

**Keywords:** Anemia, Women of reproductive age, Thatta, Factors, Perceptions

## Abstract

**Background:**

Maternal anemia is a leading public health issue placing pregnant women at higher risk of low birth weight, preterm birth, perinatal mortality, and neonatal mortality. Women in developing countries are at higher risk of anemia which could be either due to micronutrient deficiencies, hemoglobinopathies, infections or other socio-demographic factors. Thus, it is highly essential to explore the factors of anemia among women of the reproductive age group in order to design suitable interventions. The primary objective of this study is to assess the biological and socio-demographic factors that are associated with anemia among the women of the reproductive age group in Thatta district.

**Methods:**

An exploratory mixed method study using quantitative and qualitative approaches will be conducted in district Thatta Pakistan from September 2018 to January 2019. In the qualitative phase, data will be collected through focus group discussions and key informant interviews to understand the perceptions of women, their husbands and healthcare providers about anemia. In addition, a quantitative approach using cross-sectional study will be conducted to determine biological and socio-demographic factors associated with anemia. Approximately 150 non-pregnant women and their spouses will be included in the quantitative component of the study. In addition to thematic analysis for the qualitative component, Logistic regression will be done to calculate adjusted Odds ratios with their respective 95% CIs to assess the factors associated with anemia.

**Discussion:**

The better understanding of biological, socio-demographic factors and community perceptions of anemia will help us to design strategies and interventions to better address anemia during the reproductive cycle in rural areas of Pakistan. This will help the researchers and policymakers to take the appropriate action accordingly by designing suitable approaches to address the specific type of anemia in the rural population of Pakistan. This will, in turn, reduce the chances of adverse maternal and fetal outcomes associated with anemia.

## Plain English language summary

Anemia has been considered as a leading public health problem mainly among women of reproductive age group. This problem can be attributed to multiple factors such as consuming inappropriate diet, certain socio-demographic factors, susceptibility to develop some infections and hemoglobinopathies. Despite the similarities in the sociodemographic factors the prevalence of anemia widely differs between neighboring countries of India and Pakistan with high prevalence being found in rural Pakistan. Thus it is important to understand the factors associated with anemia in one of the rural districts of Pakistan. Given this condition, we propose to conduct a mixed method study using quantitative and qualitative approaches to study the biological and socio-demographic factors associated with anemia. In addition, this study will also provide an opportunity to explore the perceptions of women, their husbands and health care providers regarding anemia by conducting focus group discussions and key informant interviews. The better understanding of these factors and perceptions will help researchers and policy makers to design appropriate strategies and interventions in the rural areas of Pakistan to address the problem of anemia among women of reproductive age.

## Background

Maternal anemia is an important global health problem that affects about 500 million women of reproductive age [1]. As many as half of all pregnant women in low-income and middle-income countries are diagnosed with anemia [2, 3], which affects 32 million pregnant women worldwide [4]. Women in low-income and middle-income countries are at increased risk of anemia. This could be either due to the higher frequency of dietary micronutrient deficiencies such as iron and folic acid,, and infections such as malaria, HIV, and hookworm infestation in developing countries than developed countries [5]. Furthermore, the literature also highlights that prevalence of anemia may vary among pregnant women across low middle income countries due to differences in the lifestyles, socio-demographic and nutritional factors such as age, parity, educational and socioeconomic status, food insecurity, hygiene conditions, nutritional deficiencies and genetic predisposition that may determine the severity of anemia within the affected population [6, 7]. In addition, review of observational studies showed a linear association between maternal anemia and death, with each 10 g/l increase in maternal hemoglobin associated with a 29% reduction in maternal mortality (odds ratio [OR] 0·71 [95% CI 0·60–0·85]) [8].

WHO has recognized anemia as a global problem with serious consequence for mothers and their babies [9]. Even though anemia in pregnancy is readily treatable, data from several studies show an association between maternal anemia and severe adverse maternal and perinatal outcomes [3]. Moreover, there could be detrimental consequences of anemia during pregnancy, including the increased risks of low birth weight, preterm birth, perinatal mortality, and neonatal mortality [10].

In addition, maternal and newborn health registry (MNHR) has been maintained in 9 Union councils (UCs) of district Thatta, which documents birth outcomes and provide population-based rates of stillbirth, neonatal and maternal deaths [11, 12]. Recent MNHR data of 2017 also shows that 15.2% of pregnant women in these UCs are found to be severely anemic (Hb < 8 g. /dl), and 39.2% have been found as moderately anemic (Hb 8–10 g/dl) (Unpublished data). MNHR data also showed that severe anemia is more common in Pakistan as compared to India (0.2%) (unpublished data). Although both the India and Pakistan sites are quite similar in terms of their socio-demographic characteristics, there are huge differences in the proportion of women with very low Hb levels. Nevertheless, in both countries, the underlying cause of anemia needs to be explored across the range of socio-cultural and biological factors. Looking at these differences in the same area and with neighboring countries, it is highly essential to explore the factors of anemia among women of reproductive age group. In addition, through this study we will also explore the perceptions of women, their husbands and health care providers about anemia, its possible causes, how does it impact maternal and child health and what could be done to address this crucial problem in the district.

### Study purpose

The purpose of this study is to explore factors associated with anemia among women of the reproductive age group in Thatta district using mixed methods approach.

### Study objectives

#### The objectives of this study are two-fold

- The objective for the qualitative phase includes:
  - To explore the knowledge and perceptions of women, their husbands and health care providers about anemia, its possible causes, how does it impact maternal and child health and what could be done to address this crucial problem in the district.
- The objective for the quantitative phase include:
  - To study the sociodemographic factors associated with anemia among women of the reproductive age group in Thatta District.
  - To study the biological factors associated with anemia among women of the reproductive age group in Thatta District.
  - To study the sociodemographic factors associated with anemia among men in Thatta District.

## Methods

**Fig. 1:**
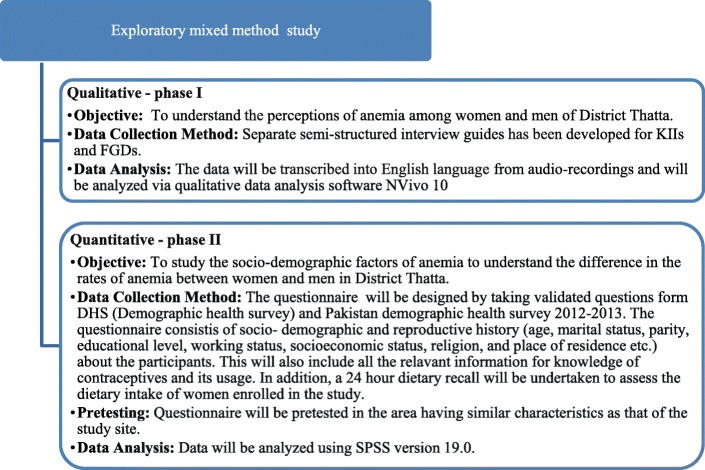
Exploratory mixed method study

This is an exploratory mixed method study using quantitative and qualitative approaches, including two study phases. In the first phase, a qualitative exploratory study will be conducted and the data will be analyzed to understand the perceptions of women, their husbands and healthcare providers regarding anemia. In the second phase, a quantitative approach using cross-sectional study will be conducted to determine factors associated with anemia. The general design of the study is presented in Fig. 1. The total duration of this substudy will be approximately 5 months (October 2018 to February 2019). The qualitative interviews will be conducted in Oct, 2018 and the data collection for quantitative phase will be from November to Dec 2018.The collected data will be analyzed and disseminated during Jan-Feb, 2018.

### Phase 1

#### Qualitative study

In this phase of the study, an exploratory qualitative research will be carried out using two semi-structured interview guides. The data collection methods for this phase will involve key-informant interviews (KIIs) and focus group discussions (FGDs). The aim of the FGDs and KIIs is to explore and understand the perceptions of women, their husbands and health care providers about anemia, its possible causes, how does it impact maternal and child health and what could be done to address this crucial problem in the district.

#### Study setting and participants

The study will be conducted at Thatta District Sindh Pakistan. Thatta is one of the rural districts of Pakistan located in the southernmost part of Sindh, bordering the two largest cities, Karachi and Hyderabad [13]. Despite its close proximity to these urban cities, Thatta was ranked 64th of 91 districts in the country on the Human Development Index in 2003 [14]. The newly formed district of Thatta now has four talukas (sub-districts) including Thatta, Mirpur Sakro, Keti Bander and Ghorabari. The Thatta district consists of 30 union councils (UCs), 257 Dehs, 3850 villages, 95,675 households (HHS) with the approximate population of 661,517, 81.6% of this population lives in rural areas of Thatta [15]. There are two public sector hospitals, 6 rural health centers (RHCs), 22 basic health units (BHUs) and 10 dispensaries in the district [16].

Despite the presence of multiple public and private schools, the total literacy rate of the district is 32.1%, where only 13% of women are literate [17]. Thatta has the lowest educational attainment score in the province and is ranked amongst the 5 lowest in the country [18]. On the other hand, Thatta does have a large number of health care providers, spread throughout the district and the proportion of women delivering at health care facilities is higher than the national average [19]. On average, 4.2 children are born to a woman during her childbearing years, while surprisingly only 10% of the women have reported using modern contraceptive methods of contraception (CPR) in the whole district [20].

The Global Network for Women’s and Children’s Health Research is the flagship research collaboration of the Reproductive Health research group in the Department of Community Health Sciences (CHS), Aga Khan University (AKU). This NICHD-funded Network is a partnership between US institutes and centers in developing countries in Asia (Pakistan and India), Africa (Kenya, the Democratic Republic of the Congo and Zambia) and Latin America (Guatemala). The Network aims to conduct multi-center trials of interventions for pregnant women, newborns, and children. In order to meet these objectives, CHS Department has maintained a Maternal Newborn Health Registry (MNHR) in Thatta District since 2008 in different geographic clusters where studies, both randomized controlled trials and cluster randomized trials of interventions to improve maternally and child health are carried out. A total population of around 170,000 is under continuous contact for research intervention.

Through MNHR, all pregnant women in the research clusters are identified registered and their outcomes are tracked for 6 weeks post-delivery. The detailed methods of the MNHR have been published elsewhere [11].

Likewise, other observational and interventional studies, MNHR provided a platform and existing research infrastructure to conduct an individually randomized controlled trial known as Woman First Trial (Preconception maternal nutrition). This trial aimed to determine the benefits to the offspring of women in poor, food-insecure environments of commencing a daily comprehensive maternal nutrition supplement ≥ 3 months prior to conception versus the benefits of commencing the same supplement at 12–14 weeks of gestation and also to compare offspring outcomes with those of a third trial arm who received no supplementation [21]. Study’s intervention included daily 20 g lipid-based (118 kcal) multi-micronutrient (MMN) supplement and, for women who were underweight or had slow gestational weight gain, an additional protein energy supplement was provided. The primary hypothesis of the study was that in women living in poor, food insecure populations, commencing a maternal nutrition supplement at least 3 months prior to pregnancy (Arm 1) would result in significantly greater fetal linear growth than starting the same nutrition supplement at 12–14 weeks gestation (Arm 2) or than not providing this supplement (Arm 3) [21]. The details of the Women First trial including design, study sites, follow-ups, various stages and ethical approvals are discussed in depth elsewhere [21].

For the current study regarding factors and perception of anemia, we propose to recruit conveniently mother-father pairs from 9 Union councils (UCs) of the district Thatta, where MNHR is currently based and subjects previously gave biological samples in Woman First (WF) trial. In addition, we will also enroll health care providers such as female and male doctors providing health services to the people of Thatta, traditional birth attendants (TBAs) who are one of the important stakeholders providing care to the women and lady health visitors or midwives who are also engaged in providing health care services to women Table 1.

**Table 1:** Number of Participants for Qualitative phase

Number of participants for the qualitative phase	Sample Range
Participants for Key-informant Interviews (KIIs)	
Healthcare providers at District Thatta (Doctors/LHVs/TBAs/Managers)	10 KIIs
Participants for Focus Group Discussion (FGDs)	
The mother who were enrolled in WF trial	5 FGDs (6–8 participants in each group)
Male (husbands) of previously enrolled mothers in WF trial	5 FGDs (6–8 participants in each group)

#### Inclusion criteria

- The study will include women and men who have previously participated in in WF trial
- The study will involve key informants’ such as Doctors, TBAs, and LHVs who are willing to participate in the study.
- Participate who are willing to give consent will be included in the study

#### Exclusion criteria

1. Women who are pregnant at the time of data collection.
2. Heath care providers, women and their husbands who do not agree to provide consent will be excluded.

#### Data collection method

Qualitative aspect will be covered by two components i.e. separate focus group discussions (FGDs) with women and their husbands and Key Informant Interviews (KIIs) with health care providers. Both of these will be conducted by developing separate guides which have been developed with the help of literature search and available expertise in the qualitative research. Prior to the actual research, the interview guide will be pretested to reveal any deficiencies in the study design.. This guide will include open-ended semi-structured questions along with probes to explore in-depth understanding about the anemia.

The questions will be exploratory in nature to encourage the free flow of information to elicit the important experiences and perceptions of the study participants regarding the women perception regarding balanced and adequate diet for a woman, anemia, risk factors and prevention and treatment of anemia. These interviews will be conducted by the principal investigator and research coordinator of the study. The interviews will be recorded after taking consent from the participants and the key points will be noted during the interview. Data collection will cease once the saturation will be achieved.

#### Data analysis

Data will be analyzed by using Nvivo 10 software. The study data gathered through FGDs and KIIs will be transcribed and translated from local language [Sindhi] to English and this would be analyzed via qualitative content analysis. The qualitative content analysis will be done to get the understanding of manifest content (what exactly transcript states) and the latent content (understanding the meaning of the transcript with context). The transcripts will be manually coded for data analysis. Transcripts will be proofread multiple times to get the correct understanding of the information provided by the participants regarding perceptions anemia. The main ideas on the transcripts will be selected and labeled as codes. Similar codes will then be analyzed and assembled into categories. In the final step subcategories with similar concepts will be grouped under the umbrella of themes. Two independent investigators to resolve the discrepancies and researchers bias will perform the coding, category formation, and thematic analysis.

### Phase 2

#### Quantitative study

In this phase of the study, a cross-sectional study will be conducted by using a structured and validated questionnaire to collect the data for this study. The questionnaire has been designed by taking validated questions form DHS (Demographic health survey) and Pakistan demographic health survey 2012–2013. The questionnaire will be consisting of socio-demographic and fertility-related information (age, marital status, parity, educational level, working status, socioeconomic status, religion, and place of residence) about the participants. This will also include all the related information for knowledge of contraceptives and its usage. Anthropometric measurements (weight, height, body mass index, waist circumference, hip circumference, mid-upper arm circumference, and head circumference.) will be conducted from enrolled women. Moreover, a 24-h dietary recall will be undertaken from enrolled women by using a 24-h dietary recall questionnaire which had also been used in the main WF trail for pregnant women in the same catchment area. This dietary recall questionnaire will help to assess the die consumed by women in district Thatta. In addition, socio-demographic characteristics of the enrolled husbands will be also collected on a separate one-page questionnaire.

The questionnaire will be pretested in the area having similar characteristics as that of the study site on the participants different from the study. The flow of questions and comprehensibility will be assessed during pre-testing the questionnaire. Refinement of the questionnaire will be done and refresher training of data collectors will be conducted by the Principal investigator and senior research coordinator of the study. This will be followed by actual and real administration of the questionnaire in the field.

#### Sample size and sampling method

A minimum sample of 150 women will be required in order to achieve a power of 80% at 5% level of significance, keeping proportion of anemia among women of reproductive age group from 21 to 75%, anticipated proportion of various risk factors in the range of 20 to 75% among anemic group and 20 to 82% among non-anemic group. In addition to 150 women, their spouses will also be included thus making a total of 300 participants to achieve the objective of quantitating phase of the proposed study. A probability simple random sampling technique will be used to select the participants. A sampling frame will be generated from existing data of WF participants and participants will be identified randomly from the given list by a third person mainly the data manager of MNHR.

#### Eligibility criteria

##### Inclusion criteria

1. Women who were enrolled in the WF trial and also allowed to collect their biological samples at 12 and 32 weeks of gestational age will be included in this study.
2. Husbands of the above-identified women will be included.

##### Exclusion criteria

1. Women who are pregnant at the time of data collection.
2. Women and their husbands who do not agree to participate in this study will be excluded.

### Data collection method

We are proposing to recruit from mother-father pairs of subjects previously enrolled and gave biological samples in WF. New samples to be collected in the proposed sub-study include blood samples and stool samples only from women of previously enrolled WF participants. Around 12–15 mL of venous blood will be collected by the trained and professional phlebotomist. Blood samples will be used to do the investigations such as complete blood count (CBC), the presence of the malarial parasite, Iron levels, Erythrocyte Sedimentation Rate (ESR), C-Reactive Protein Test (CRP), B-12 and folic acid levels and identification of hemoglobinopathies through hemoglobin (Hb) electrophoresis. In addition, the hemoglobin of enrolled women will also be checked by using the hemocue machine to be consistent with WF primary study methods and to see the differences between hemoglobin levels being measured by taking venous blood and by making finger prick. Likewise, stool samples will be used to assess the worm infestation. In this study, no biological sample will be collected from husbands of the participating women; except the hemoglobin levels which will be checked by using hemocue machines and a small questionnaire will be administered to husbands to collect the data on their socio-demographic factors.

### The detailed procedure for data collection

The home visitor research assistant (HVRA) responsible for collecting the data for WF primary trail will be trained in the proposed study to administer new questionnaires while visiting their households. In the field, HVRA will discuss the purpose of the study with the whole family and will also assess the eligibility of woman before taking consent from her. In order to check the pregnancy status, HVRA will take a brief history of last menstrual period and symptoms of pregnancy in the local language. In addition, she will also perform a pregnancy urine test to confirm the pregnancy status of the woman. A woman will be excluded from the study if a positive pregnancy test is found and will be included in the study otherwise. After woman understands the procedures of study completely, HVRA will take a written informed consent from a woman. Once informed consent is obtained, the HVRAs will 1) administer the relevant questionnaires, 2) conduct anthropometric measurements (weight, height, body mass index, waist circumference, hip circumference, mid-upper arm circumference, and head circumference.) and 3) will also facilitate phlebotomist to collect the biological samples at different collection points of AKU. At the end of the questionnaire, a section will be provided for the data collectors to note down any important information they got during the interview. The questionnaire will have the interviewer’s name mentioned on it as well as the date of the filling of the form and information about the contact number of the investigator and the contact number and/or the address of the respondent, so that any queries at the field or anywhere in between the study can be addressed in an efficient manner.

In addition, male community mobilizers will contact the husbands of the eligible women in order to enroll them in the study. Likewise, male community mobilizers will approach to husbands of identified women and will ask some information related to their socio-demographic factors, personal habits, and daily routines by administering a structured and pre-tested questionnaire. Like HVRAs, male community mobilizers will exactly follow the same procedure of taking written informed consent from husbands.

Our trained HVRAs and male community mobilizers are native speakers of the local languages and are familiar with the culture will carry out consenting and procedures in the local language. For those participants who are illiterate, a family member of their choice or a member of the community who is not part of the research staff will serve as a witness. The HVRAs and male community mobilizers will explain the study and allow the family to ask any questions about it and to discuss it among themselves. Comprehension will be attested by asking the participant to describe the study in their own words. If the family would like to continue with the data/sample collection, they will be asked for written informed consent in the local language.

### Biological sample collection, transportation, and analysis

Two types of biological samples (blood and stool samples) will be collected from enrolled women in the proposed study. There are three different blood collection points in the catchment area of MNHR in district Thatta where samples will be collected. HVRAs will inform women at least 1 day before to collect biological samples from them and they will fix an appointment with them as per their availability and convenience. After informing women, HVRAs will visit women at their homes and will bring them to the blood collection points of AKU. Each sample collection is completely voluntary and can be denied at any time. Written informed consent will be obtained separately before any sample collection occurs.

At the blood collection point, a trained and qualified phlebotomist will greet the woman and explain the procedures of blood collection to her. The phlebotomist will collect blood samples from the woman by following universal precautions and standard procedures of AKU laboratory. A unique number and barcode will be assigned to each sample being collected from the woman and relevant information such as name and unique WF ID of woman and names of investigations (CBC, iron, B12 etc.) will be entered into the computer on the spot. The woman can submit the stool sample on the same day or she will be guided by HVRA to give stool sample the next day. A complete kit including clean sheet, gloves, Coleman box with ice packs and stool container with scoop will be provided to every woman to pass and store stool sample in a clean area on appropriate temperature. HVRA will contact the woman on the next day to inquire if she has passed stool sample and will collect the sample from woman to submit the same on appropriate collection point. This whole procedure of data collection along with biological sample collection will take about 60–90 min to complete the data collection from each individual participant.

All biological sample being collected from enrolled women will be transferred from Thatta collection points to the main Aga Khan Hospital Karachi Pakistan under the required cold chain. The main laboratory of the Aga Khan University Hospital Karachi is well equipped and has the capacity to analyze the blood samples for the proposed markers and investigations such as complete blood count (CBC), serum iron, folic acid, Erythrocyte Sedimentation Rate (ESR), C-Reactive Protein Test (CRP), B12, ferritin, hemoglobinopathies, the presence of malarial parasite and stool analysis for worm infestation. Biological samples will be stored and analyzed in the Aga Khan Hospital laboratory by qualified personnel. The remaining samples will be destroyed as per AKU laboratory policy at the end of the study mainly after the analysis is done and reports are generated. Once the laboratory reports are generated, HVRAs will hand over reports to the women and will advise and facilitate them to contact local health care provider for getting health services free of cost from district hospital if required.

### Data management and quality assurance

#### Data monitoring and quality assurance

Regular supervision of the HVRAs in the respective geographic areas will be conducted by the field supervisors who are experienced and have been working in the catchment areas for the last ten years. Each field supervisor will be assigned five geographic areas to make supervisory and monitoring visits and to provide on jo coaching to HVRAs. These field supervisors will review the filled questionnaire to ensure that all sections of the questionnaire are filled properly and consistency checks with skip patterns have been followed correctly before leaving the home of the participant. In addition, Principal investigator (PI) and research coordinator (RC) of the study will also review forms and make surprise visits to monitor the data collection and to guide HVRAs and field supervisors whenever necessary. These filed supervisors will also update PI about the daily progress of the study via phone calls and by sending a summary report on the WhatsApp group generated for this study.

Quality of data collection will be ensured by proper training of the HVRAs and field supervisors. Quality will also be ensured through supervision and random inspection of the data collection process by the principal investigator or senior research coordinator in the field. In addition, meetings will be arranged to maintain coordination among the members of the team and to keep the members up-to-date and to resolve any field related issues.

#### Data editing

Data editing will be done in two phases i-e field editing and office editing. Field editing will be done by the field supervisors where they will check each questionnaire filled by HVRA on the hard copy of questionnaire every week. These filed supervisors will hand over forms to quality assurance officer on a weekly basis who will bring forms to AKU data management unit and will edit forms in the central office of AKU.

#### Data entry and cleaning

Two data entry operators will enter the data into a data entry program in parallel. The data entry program will be designed using EPI data. Data entered will be compared for entry errors and the identified errors will be corrected. 10% of the records will be randomly chosen from the database and will be validated against the questionnaire data. If there is more than 0.3% discrepancy between the two, data entry will be done again. The hard copy forms will be kept in the secure location for possible once the double data entry is completed.

#### Data analysis

Quantitative data will be double-entered in EPI-Data software. The primary outcome of analysis will be anemia which has been defined in agreement with WHO definitions for anemia, which are Hb < 12.0 g/dl or Hct < 36% for non-pregnant women [22]. Frequencies and percentages will be calculated for qualitative variables like working status, education level, and employment status, the occupation of the woman and her husband, any pre-existing medical conditions. Means and standard deviations or median with inter-quartile range whatever will be appropriate for the continuous variables including current age of the woman, age at the time of marriage, parity, number of alive children and number of prior sons etc. will be generated for all participants.

Univariate analysis will be conducted by computing unadjusted odds ratio (OR) and their 95% confidence intervals to compare the anemic and non-anemic women with respect to all the possible biological and socio-demographic factors. The appropriate test for example chi-square will be used to assess the association between variables. Multicollinearity between independent variables will be assessed. The variables for which *p*-value turns out to be less than 0.25 will be included in the multivariable analysis. In the multivariable analysis, multiple logistic regressions will be used to compute the adjusted odds ratio with 95% confidence intervals. During this analysis interaction between the variables will be assessed. This analysis will also be helpful in identifying the potential confounders related to the study. Data will be analyzed using SPSS version 19.0 version.

### Ethical considerations

#### Ethical approval

This study will be conducted only after the affirmative decision that the study had been reviewed and could be conducted at the identified sites by an ethical review committee (ERC) of the Aga Khan University.

#### Informed consent

Important ethical considerations include the decision of the participant in the study after informing and making her understand comprehensively the objectives of the study. Written informed consent will be taken from each participant prior to enrolling them in the study. Women who are unable to read and write will be informed in detail about the study and thumb printing will be taken from them instead of the signature.

#### Privacy and confidentiality

Since the data will also be collected on certain sensitive issues, like contraceptives methods and its, therefore, information from the participant will be taken by maintaining the privacy and confidentiality of the participant. A particular ID will be issued to each participant so that the names of any participant might not be disclosed. All data forms, reports, and other records that leave the site will be identified by coded number only to maintain participant confidentiality. All records will be kept in a secure location and will be shared only with those who will be directly involved in the study such as PI and research coordinator. All computer entry and networking programs will be done with coded numbers only.

## Discussion

The better understanding of biological, socio-demographic factors and community perceptions of anemia will help us to design strategies and interventions to better address anemia during the reproductive cycle in rural areas of Pakistan. This will, in turn, reduce the chances of adverse maternal and fetal outcomes associated with anemia. Moreover, this study will also help us to identify the type of anemia such as iron deficiency anemia, megaloblastic anemia or anemia due to hemoglobinopathies. This will help the researchers and policymakers to take the appropriate action accordingly by designing suitable approaches to address the specific type of anemia in the rural population of Pakistan. Thus, the benefits of this study have the potential to impact the general population by enhancing our understanding of what factors contribute to anemia and what can be done to improve the health of women of reproductive age in District Thatta. In addition, the qualitative component of the study will help us to understand the perception and knowledge of community people and health care providers regarding anemia. This will help researchers and policymakers to have an in-depth understanding of opinions of the community regarding anemia in the rural population.

## Abbreviations

CHS: Community Health Sciences
ERC: Ethical review committee
FGDs: Focus group discussions
HVRA: Home visitor research assistant
IDIs: In-depth interviews
LHV: Lady health visitor
MNHR: Maternal and newborn health registry
TBA: Traditional birth attendant
UCs: Union councils
WF: Women first
WHO: World Health Organization
